# Heterostructured CoO_x_–TiO_2_ Mesoporous/Photonic Crystal Bilayer Films for Enhanced Visible-Light Harvesting and Photocatalysis

**DOI:** 10.3390/ma13194305

**Published:** 2020-09-26

**Authors:** Stelios Loukopoulos, Alexia Toumazatou, Elias Sakellis, Evangelia Xenogiannopoulou, Nikos Boukos, Athanasios Dimoulas, Vlassis Likodimos

**Affiliations:** 1 Section of Condensed Matter Physics, Department of Physics, National and Kapodistrian University of Athens, 15784 Panepistimiopolis, Greece; sloukop@phys.uoa.gr (S.L.); alextoum@phys.uoa.gr (A.T.); 2 Institute of Nanoscience and Nanotechnology, National Center for Scientific Research “Demokritos”, Agia Paraskevi, 15341 Athens, Greece; e.sakellis@inn.demokritos.gr (E.S.); e.xenogiannopoulou@inn.demokritos.gr (E.X.); n.boukos@inn.demokritos.gr (N.B.); a.dimoulas@inn.demokritos.gr (A.D.)

**Keywords:** photonic crystals, titanium dioxide, cobalt oxides, visible light, photocatalysis, slow photons

## Abstract

Heterostructured bilayer films, consisting of co-assembled TiO_2_ photonic crystals as the bottom layer and a highly performing mesoporous P25 titania as the top layer decorated with CoO_x_ nanoclusters, are demonstrated as highly efficient visible-light photocatalysts. Broadband visible-light activation of the bilayer films was implemented by the surface modification of both titania layers with nanoscale clusters of Co oxides relying on the chemisorption of Co acetylacetonate complexes on TiO_2_, followed by post-calcination. Tuning the slow photon regions of the inverse opal supporting layer to the visible-light absorption of surface CoO*_x_* oxides resulted in significant amplification of salicylic-acid photodegradation under visible and ultraviolet (UV)–visible light (Vis), outperforming benchmark P25 films of higher titania loading. This enhancement was related to the spatially separated contributions of slow photon propagation in the inverse opal support layer assisted by Bragg reflection toward the CoO*_x_*-modified mesoporous P25 top layer. This effect indicates that photonic crystals may be highly effective as both photocatalytically active and backscattering layers in multilayer photocatalytic films.

## 1. Introduction

Shaping photocatalytic materials in the form of photonic crystals (PCs) has become a competent structural modification that exploits the unique capabilities for slow photon-assisted light harvesting, pore interconnectivity, and surface accessibility of periodic macroporous structures, such as inverse opals [1]. These distinctive structural features can be effectively combined with rational compositional tuning of the materials’ properties for charge separation and visible-light activation (VLA) leading to efficient visible-light photocatalysts [2,3]. The underlying amplification is based on photonic band gap (PBG) engineering, which enables overlap of the slow photon spectral regime with the catalyst’s electronic absorbance resulting in a longer optical path for incident photons within the porous PC network [4,5]. However, in addition to single-layer, monolithic inverse opals, PCs have also attracted attention as photocatalytically inert supports for thin-film photocatalysts, where their strong PBG (Bragg) reflection is tuned to the catalysts’ electronic absorption edge [6,7,8,9,10]. This effect was originally applied to enhance the light-harvesting efficiency of dye-sensitized solar cells by coupling a TiO_2_ inverse opal layer on a conventional nanoparticulate titania photoelectrode [11], exploiting the back reflection in the PBG region of the PC layer that acts as a dielectric mirror and the excitation of localized slow photon modes within the nanocrystalline mesoporous film [12,13].

In the case of PC supporting layers for photocatalytic films, light transmitted through the photocatalytically active top layer is selectively backscattered by the PC substrate according to its PBG, which can be adjusted to match the targeted spectral region of the photocatalyst’s electronic absorbance. Multifold improvements of the photocatalytic performance were, thus, reported for C_3_N_4_ nanorod [6], TiO_2_ nanoparticulate [7,10], and CdS quantum dot [8] films supported on SiO_2_ opals, as well as for CO_2_ photomethanation over dispersed RuO_2_ nanocrystals on Si inverse opals [9]. Despite the advantages of PC-supported photocatalysts, the prospect of using a photocatalytically active PC substrate as a means to combine visible-light trapping and Bragg reflection has hardly been explored for thin film photocatalytic applications.

Surface modification of TiO_2_ by metal-oxide nanoscale clusters has emerged as a facile yet very efficient method for the development of visible-light-activated (VLA) photocatalysts for solar-driven applications including organic pollutant decomposition and water splitting [14,15,16,17,18,19,20]. Among wet impregnation routes of different metal salt precursors, chemisorption of the bulky metal acetylacetonate complexes on titania followed by post-thermal decomposition has been an effective approach for the dispersion and controlled loading of molecular-scale metal-oxide nanoclusters on TiO_2_ [21]. Broadband visible-light activation of TiO_2_ nanoparticles was related to the formation of metal–O–Ti interfacial bonds, generating surface states that raise titania’s valence band without introducing intragap defect states of low oxidation potential [22]. Among the different metal oxocomplex surface-modified titania catalysts, CoO*_x_*-TiO_2_ presented the highest performance among nanoparticulate systems for the degradation of organic pollutants under visible and UV light [23] and water oxidation [17,18,19,20].

In this work, bilayer films consisting of a TiO_2_ PC bottom layer and a highly reactive titania top layer, surface-modified by CoO_x_ nanoclusters as VLA sensitizers, are demonstrated as efficient photocatalytic films that feature enhanced visible-light harvesting and improved photocatalytic activity by means of slow photon propagation and Bragg backscattering. To this aim, well-ordered PBG engineered anatase TiO_2_ inverse opal underlayers were deposited via the evaporation-induced coassembly of PMMA colloidal spheres of different diameters with a hydrolyzed Ti alkoxide precursor, while a mesoporous TiO_2_ overlayer was deposited using the benchmark Aeroxide^®^ P25 titania catalyst, leading to bilayer films with smooth interfaces. Tuning the PBG/slow photon regions of the PC substrates to the visible-light electronic absorption of the surface CoO_x_ oxides resulted in the enhancement of salicylic acid’s photocatalytic degradation, outperforming benchmark mesoporous P25 films of higher thickness. The optimal performance was achieved in the case of slow photon amplification for the TiO_2_ PC bottom layer in combination with a smaller contribution by Bragg reflection to the P25 top layer, indicating that photonic crystals can be very effective as both photocatalytically active layers and Bragg mirrors in heterostructured photocatalytic films.

## 2. Materials and Methods

### 2.1. Bilayer Film Deposition and Surface Modification

**Scheme 1:**
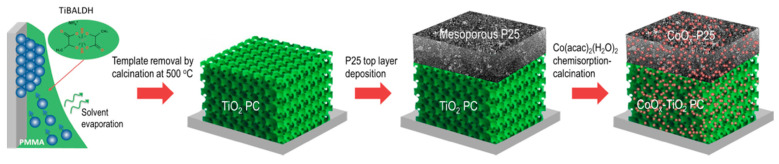
Schematic illustration of the CoO_x_-P25/photonic crystal (PC) bilayer deposition process.

TiO_2_ inverse opal bottom layers were deposited on glass substrates in a single-step process via the evaporation-induced coassembly [24,25] of monodisperse poly(methyl methacrylate) (PMMA) spheres (Microparticles GmbH, Berlin, Germany) in the form of 5% solid (*w*/*v*) colloidal dispersion in deionized water (2.0–2.3% CV) and a hydrolyzed Ti alkoxide precursor through a titanium(IV) bis(ammonium lactato)dihydroxide (TiBALDH) 50 wt.% aqueous solution (388165, Sigma-Aldrich, St. Louis, MO, USA). In a typical deposition, clean glass slides were nearly vertically suspended in 10 mL of a 0.125 wt.% PMMA sphere suspension and 0.07 mL of fresh precursor prepared by sonication of 0.25 mL of TiBALDH solution, 1 mL of EtOH (32221, Honeywell Riedel-de Haën, puriss. p.a., absolute, ≥99.8%), and 0.5 mL of 0.1 M HCl (30721 Fluka, ACS reagent, fuming, ≥37%). The vials were kept at 55 °C until the solvent fully evaporated, yielding composite films comprising the titania gel within the opal interstices. The dry films were calcined at 500 °C for 2 h in air (1 °C/min), to remove the PMMA matrix and crystallize TiO_2_ in the inverse structure (Scheme 1).

PMMA spheres of 406 nm (PMMA-R-KM290, Microparticles GmbH, Berlin, Germany) and 499 nm (PMMA-R-KM296, Microparticles GmbH, Berlin, Germany) diameters were used to deposit the PC films, labeled as PC406 and PC499, respectively. Mesoporous TiO_2_ layers were deposited on top of the PC films by spin-coating (1000 rpm for 60 s) Aeroxide^®^ P25 titania paste [26] followed by drying (120 °C) and post-annealing at 450 °C (Scheme 1). The P25 layer deposition was repeated two times on the PC substrates, leading to bilayer films labeled P25/PC406 and P25/PC499. The layer thickness was selected according to photocatalytic screening tests showing that a single P25 layer and/or thinner PCs, for which the photonic properties are not fully developed, led to reduced photocatalytic activity. In order to validate the bilayer’s performance, a thick benchmark P25 reference film, designated as P25t, was deposited by successive spin-coating cycles on glass slides. Surface modification by Co oxides (CoO*_x_*) was performed via the chemisorption of cobalt(II) acetylacetonate dihydrate Co(acac)_2_(H_2_O)_2_ (C0373, TCI, Tokyo, Japan) on the titania films after immersion in 100 mL of a 10^−3^ Μ solution (3:17 *v/v* EtOH/*n*-hexane) for 24 h followed by thorough washing with the same solvent, drying, and post-calcination in air at 500 °C for 1 h [22]. The corresponding monolithic and bilayer films were designated as CoO_x_-PC406, CoO_x_-PC499, CoO_x_-P25, CoO_x_-P25/PC406, CoO_x_-P25/PC499, and CoO_x_-P25t.

### 2.2. Material Characterization

The film morphology and phase composition were studied using a scanning electron microscope (SEM, Quanta Inspect, FEI, Eindhoven, Netherlands) coupled with energy-dispersive X-ray spectroscopy (EDXDX4, EDAX, Mahwah, NJ, USA) and a FEI Talos F200i field-emission (scanning) transmission electron microscope (Thermo Fisher Scientific Inc., Waltham, MA, USA) operating at 200 kV, equipped with a windowless energy-dispersive spectroscopy microanalyzer (6T/100 Bruker, Hamburg, Germany). The structural properties of the films were characterized by micro-Raman spectroscopy (inVia Reflex, Renishaw, London, UK) at 785 and 514.5 nm excitations. The laser beam was focused though a pinhole using a 100× (ΝA = 0.9) objective at low power density (0.1 mW/μm^2^) to avoid local heating. The Co oxidation state was analyzed by X-ray photoelectron spectroscopy (XPS) on a PHOIBOS 100 (SPECS, Berlin, Germany) hemispherical analyzer using a monochromatic Al Kα X-ray source (1486.6 eV). The spectrometer was calibrated using clean silver copper and gold samples. The Ag *3d*_5/2_, Cu *2p_3_*_/2_, and Au *4f*_7/2_ peak positions were determined at 368.3, 932.7, and 84 eV, respectively. The XP spectra were collected at 38° using a pass energy of 10 eV. The optical properties were investigated by specular and diffuse reflectance UV–visible light (Vis) spectroscopy (Cary 60 UV–Vis, Agilent, Santa Clara, CA, USA) equipped with fiber-optic diffuse reflectance (Barrelino) and 15° specular reflectance (PIKE, UV–Vis 15Spec, PIKE Technologies, Madison, WI, USA) accessories, using a Halon standard and a UV-enhanced Al mirror for baseline measurements.

### 2.3. Photocatalytic Performance

The photocatalytic activity of single and bilayer films was evaluated on the photodegradation of salicylic acid (SA, 247588, Sigma-Aldrich) under UV–Vis and visible light. The films (~1.5 cm^2^) were placed horizontally in a vial containing aqueous SA (4 mL, 25 μΜ) solution, where they were left for 60 min under dark conditions and stirring to reach adsorption–desorption equilibrium. To enhance SA adsorption on the TiO_2_ films, the solution pH was stabilized at 3 by dilute HCL [27,28]. UV–Vis irradiation was provided by a 150 W Xe lamp (6255, ORIEL GmbH, Darmstadt, Germany) along with a long-pass edge filter with 305 nm cut-on (20CGA-305, Newport, RI, USA) and a heat-reflective mirror (20CLVS-3 CoolView™, Irvine, CA, USA). Visible light was selected by an additional long-pass filter with 400 nm cut-on (20CGA-400, Newport, RI, USA). The horizontal beam was directed on the film surface by a UV-enhanced Al mirror (ValuMax 20D520AL.2, Newport, RI, USA). The incident power density on the film surface was 76 mW/cm^2^. A 0.5 mL aliquot was periodically withdrawn from the SA solution and analyzed in a 10 mm quartz micro cell (105B-QS, 500 μL, HELMA Analytics, Munich, Germany) in the Cary 60 spectrophotometer. The photocatalytic experiments were performed in triplicate, and standard errors were calculated for the mean kinetic constants.

## 3. Results and Discussion

### 3.1. Morphological, Structural, and Optical Properties

**Figure 1:**
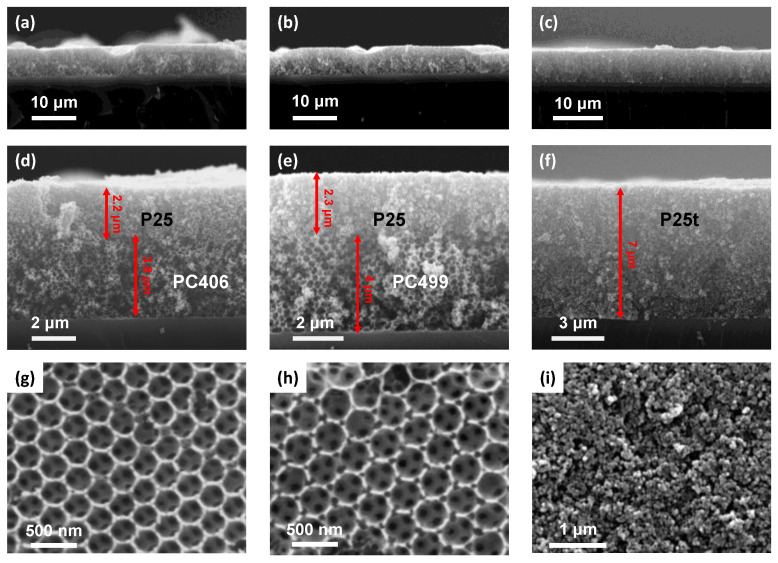
Cross-section SEM images of (**a**,**d**) CoO*_x_*-P25/PC406, (**b**,**e**) CoO_x_-P25/PC499, and (**c**,**f**) CoO*_x_*-P25t films at different magnifications. Top-view images of (**g**) CoO*_x_*-PC406, (**h**) CoO*_x_*-PC499, and (**i**) P25 layers.

Cross-section SEM images of the CoO*_x_*-P25/PC bilayer and the reference CoO_x_-P25t films at successively higher magnifications, displayed in Figure 1a–f, show the formation of smooth interfaces between the ~2.2 μm thick P25 top layer and the coassembled ~4 μm thick PC support. Top-view SEM images of the constituent layers, Figure 1g–i, verify the deposition of well-ordered inverse opals consisting of the (111) planes of an *fcc* lattice of interconnected void macropores with mean diameters of 255 and 310 nm for PC406 and PC499, respectively, in contrast to the rough morphology of the mesoporous P25 films. The interconnected P25 aggregates inhibited any significant infiltration of the mesoporous top layer to the PC substrate, most clearly seen for the CoO_x_-P25/PC499 bilayer with the larger macropores (Figure 1e). The P25t film thickness reached ~7 μm, leading to increased titania mass loading, a least twofold higher than that of the bilayer films for ideal (26%) filling of the inverse opal space.

**Figure 2:**
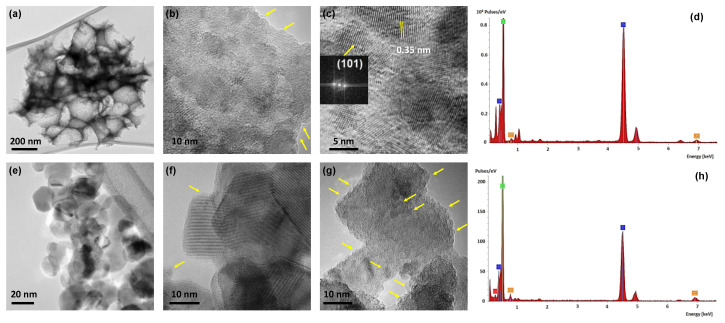
TEM images at different magnifications for the (**a**–**c**) CoO*_x_*-PC406 and (**e**–**h**) CoO*_x_*-P25 films. (**d**,**h**) Energy-dispersive X-ray (EDX) spectra corresponding to the areas of the TEM images of (**b**,**f**).

Figure 2a–c present characteristic TEM images of the surface-modified PC films showing the formation of mesoporous walls that consist of aggregated anatase nanocrystallites (<10 nm), identified by the most common (101) anatase planes (*d* = 0.35 nm), as depicted by the fast Fourier transform (FFT) pattern of the indicated nanoparticle in the inset of Figure 2c. Small protrusions of 1–2 nm could be barely discerned at the edges of the anatase nanoparticles, as shown in Figure 2b, with no distinct FFT pattern due to crystalline Co oxides, in agreement with the presence of ultrafine CoO*_x_* species.

This was directly evidenced by EDX analysis of TEM images such as that of Figure 2b, where the CoK peak was clearly detected, as shown in Figure 2d. The growth of CoO*_x_* nanoclusters can be accordingly inferred on the anatase inverse opal walls, complying with the “molecular” CoO*_x_* clusters anticipated by the chemisorption–calcination of Co(II) acetylacetonates on titania [14,23]. Likewise, well-crystallized anatase nanoparticles on the order of 20 nm, along with larger rutile nanocrystals, could be invariably identified in the TEM images of CoO*_x_*-P25, shown in Figure 2e–g. Dark surface deposits of 2–3 nm could be also discerned to decorate the titania nanoparticles, corroborating the grafting of CoO_x_ nanoclusters on TiO_2_ for the CoO*_x_*-P25 films [18,29]. The presence of Co species was directly verified by local EDX analysis, as shown in Figure 2h.

**Figure 3:**
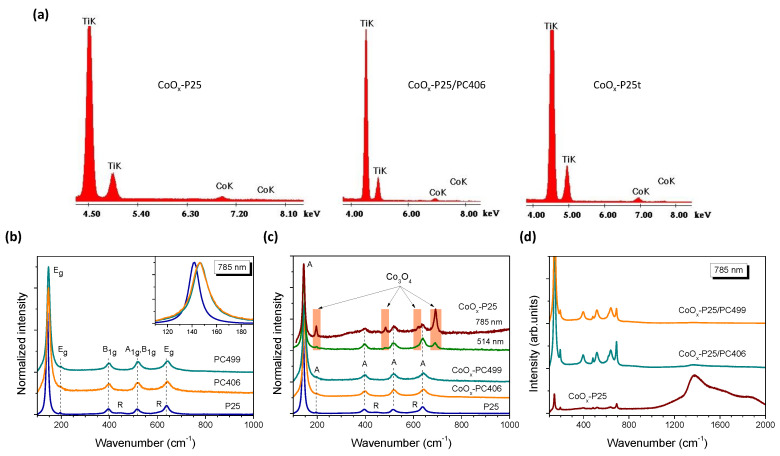
(**a**) Representative EDX spectra of the surface-modified CoO_x_-TiO_2_ films. Raman spectra of monolithic PC and P25 films (**b**) before and (**c**) after CoO_x_ deposition. The inset in (**b**) compares the corresponding low-frequency anatase E_g_ modes, while arrows and shaded bands in (**c**) indicate the Co_3_O_4_ spinel modes. (**d**) Comparative Raman spectra for the CoO_x_-P25 and bilayer films at 785 nm.

The loading amount of Co species on the surface-modified films was quantitatively determined by EDX analysis carried out in the SEM instrument on the basis of Ti K and Co K peaks detected over the whole film area, as shown in Figure 3a. The values of 2.76, 2.40, 3.11, and 2.78 Co at.% were obtained for the CoO*_x_*-P25/PC406, CoO*_x_*-P25/PC499, CoO*_x_*-P25, and CoO*_x_*-P25t films, respectively, indicating a slightly higher uptake of CoO_x_ clusters by the mesoporous P25 films comprising larger titania nanoparticles. The structural properties and phase composition of the CoO_x_-TiO_2_ films were further studied by Raman spectroscopy. Figure 3b compares the Raman spectra of the individual PC and P25 films. The inverse opals presented the characteristic Raman-active phonons of anatase TiO_2_ at approximately 147 (E_g_), 197 (E_g_), 398 (B_1g_), 518 (A_1g_ + B_1g_), and 642 cm^−1^ (E_g_). No traces of PMMA or other TiO_2_ polymorphs such as rutile or brookite were detected, confirming that the PC films crystallized in the single anatase phase after calcination at 500 °C. The anatase modes, especially the low-frequency E_g_ one depicted in the inset of Figure 3b, presented significant shifts and broadening for the PC films compared to P25, where the main rutile modes could be also detected. This variation is characteristic of the breakdown of the *q* = 0 selection rule for Raman scattering and the concomitant size effects in the Raman spectra of titania nanomaterials [30]. The formation of ca. 8 nm anatase nanoparticles can be predicted from the frequency and width of the E_g_ mode [31] in agreement with the TEM results, corroborating the formation of small-size (≤10 nm) anatase nanocrystals in the coassembled TiO_2_ inverse opals using the TiBALDH precursor [25].

The Raman spectra for the surface-modified titania films revealed the preferential Co_3_O_4_ formation for the CoO_x_-P25 films, as shown in Figure 3c. In that case, in addition to the anatase and rutile Raman bands, the characteristic phonons at 194, 483, 619, and 692 cm^−1^ of the Co_3_O_4_ spinel phase were observed [32]. The intensity of the spinel Raman modes were significantly enhanced at 785 nm, compared to the corresponding Raman spectrum at 514.5 nm, because the near-infrared laser excitation energy approached the strong electronic absorption due to the metal-to-metal charge transfer transition of Co_3_O_4_ near 750 nm [33] and the resultant resonance Raman effect.

On the other hand, the Co_3_O_4_ bands were not detected for the CoO_x_-PC films, despite the enhanced Raman sensitivity to minute spinel deposits at the near resonant excitation of 785 nm. The Co_3_O_4_ Raman modes were similarly identified for the CoO*_x_*-TiO_2_ bilayer films due to the spinel formation in the P25 top layers, as shown in Figure 3d. In that case, the parasitic glass fluorescence (1200–1600 cm^−1^), which is dominant in the wide-range Raman spectra of the monolithic CoO_x_-P25 films, decreased due to the intervention of the PC bottom layer on the glass substrate. On the other hand, the formation of single-valence CoO and Co_2_O_3_ oxides, which present weak Raman scattering, is very likely, especially for the supporting PCs, where similar amounts of Co loading to the P25 films were detected by EDX. In particular, only weak second-order and defect-induced Raman scattering is allowed for the centrosymmetric rock-salt structure of CoO [34], whereas the less stable Co_2_O_3_ phase presents similar Raman spectra to Co_3_O_4_ [35]. This implies that their formation with low crystallinity or the presence of amorphous CoO*_x_* is very probable even for the mesoporous P25 layers. This would conform with recent results by X-ray photoelectron spectroscopy on surface-modified P25 nanoparticles by the wet impregnation of Co(acac)_3_·3H_2_O, which indicated the presence of both Co^2+^ and Co^3+^ ions in CoO*_x_*-TiO_2_, in the form of oxides, mixed oxides, or hydroxides, and even metallic Co species [18].

**Figure 4:**
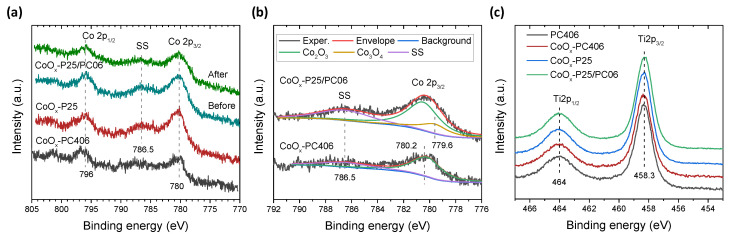
(**a**) Co *2p* X-ray photoelectron spectra (XPS) of CoO_x_-TiO_2_ films. The XPS after the photocatalytic experiment is included for CoO*_x_*-P25/PC406. (**b**) Deconvolution of the Co *2p*_3/2_ spectra for CoO*_x_*-PC406 and CoO*_x_*-P25/PC406. (**c**) Comparative Ti *2p* spectra for pristine and surface-modified CoO*_x_*-TiO_2_ films.

The Co oxidation state was investigated by XPS measurements. Figure 4a compares the XP Co *2p* core level spectra for the bilayer CoO*_x_*-TiO_2_ films and their constituent single-layer films. In all cases, a broad Co *2p*_3/2_ peak was observed at a binding energy of approximately 780 eV along with a broad Co *2p*_1/2_ signal at about 796 eV, indicating primarily the formation of Co_2_O_3_ oxides [36]. Spectral deconvolution of the Co *2p*_3/2_ region showed a dominant peak at 780.2 eV accompanied by a broad satellite (SS) peak at 786.5 eV separated by ΔS ~6.5 eV from the main Co *2p*_3/2_ peak. This value corroborates the preferential formation of Co_2_O_3_ for which ΔS ~6.3 eV rather than Co_3_O_4_ (ΔS ~7.5 eV) or CoO (ΔS ~7.9 eV) [23,36]. In addition, a weak shoulder to the Co *2p*_3/2_ peak was identified at 779.6 eV for the films comprising a CoO_x_-P25 layer. This implies that Co_3_O_4_ spinel formation is also likely, complying with the results of Raman analysis. A mixed Co oxidation state can, thus, be inferred for the CoO_x_-TiO_2_ bilayer films in agreement with recent results on surface-modified P25 nanoparticles [18] and monolithic PC films [37] by Co acetylacetonate complexes.

Figure 4c shows representative Ti *2p* spectra for pristine and CoO_x_-TiO_2_ films, where the characteristic Ti *2p* spin-orbit doublet was observed. The *2p*_3/2_ and *2p*_1/2_ peaks were centered at 458.3 and 464 eV, respectively, with a separation of 5.7 eV, confirming the presence of Ti^4+^ ions and stoichiometric TiO_2_ [38], independently of the CoO*_x_* deposition. It should also be noted that all films exhibited similar C *1s* signals due to adventitious carbon that varied weakly after CoO*_x_* deposition or PMMA template removal, indicating that organic residues in the film pores are rather low after thermal treatment in air at 500 °C. This is in agreement with recent TGA analysis of CoO*_x_*-P25 nanoparticles [18], where thermal decomposition of acetylacetonate species was found to occur at lower temperatures compared to the neat precursor, leading to the selection of 350 °C for 10 h as optimum treatment conditions.

### 3.2. Optical Properties

**Figure 5:**
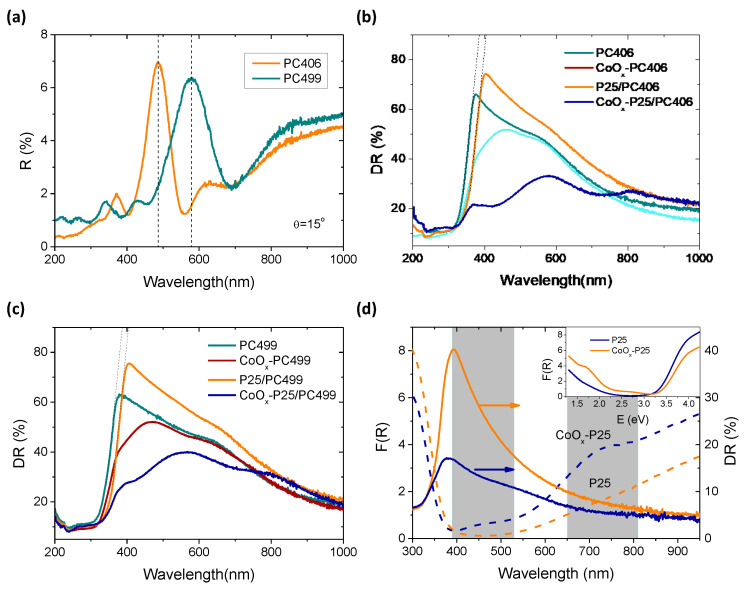
(**a**) Specular reflectance (R%) spectra for the PC406 and PC499 films. Diffuse (DR%) reflectance spectra for the pristine and CoO_x_-modified single- and bilayer films supported on the (**b**) PC406 and (**c**) PC499 inverse opals. (**d**) The DR% and the corresponding Kubelka–Munk absorbance spectra for the P25 and CoO_x_-P25 films. The inset shows the corresponding F(R) spectrum in eV.

The PBGs of the titania inverse opals were identified at 486 and 578 nm from the specular reflectance (R%) spectra for the PC406 and PC499 films, as shown in Figure 5a. The PBG positions can be approximately described by the modified Bragg’s law for first-order diffraction from the (111) planes of the *fcc* inverse opal structure [2].

(1) $$\lambda =2d_{111}\sqrt{n_{eff}^{2}-\sin^{2}\theta }$$

where λ is the stop band wavelength, $d_{111}=\sqrt{\frac{2}{3}}\;D$ is the spacing between the (111) planes, *D* is the macropore diameter, and $n_{eff}^{2}=n_{\mathrm{sphere}}^{2}f+n_{{\mathrm{TiO}}_{2}}^{2}\left(1-f\right)$ is the volume-weighted average of the sphere’s refractive index $\left(n_{\mathrm{sphere}}\right)$ and titania $\left(n_{{\mathrm{TiO}}_{2}}\right)$, while *f* is the sphere-filling fraction (*f* = 0.74 for the *fcc* lattice), and *θ* is the angle between the incident beam and the (111) direction. Using the experimental PBG wavelengths at *θ* = 15° and the measured sphere diameters for $n_{{\mathrm{TiO}}_{2}}=2.55$ and $n_{\mathrm{sphere}}=n_{\mathrm{air}}=1.0$, the $n_{eff}$ values of 1.20 and 1.17 and the titania filling fractions (1 − *f*) of 0.078 and 0.067 for PC406 and PC499, respectively, were determined in air. The latter values were much smaller than the theoretical one of 0.26 for complete filling of the inverse *fcc* lattice, supporting the mesoporosity of the nanocrystalline anatase skeleton. Furthermore, using the obtained (1 − *f*) values for $n_{\mathrm{sphere}}=n_{H_{2}O}=1.33$, the PBGs were calculated at 609 and 731 nm for PC406 and PC499 at 0° incidence in water, where the photocatalytic reaction takes place.

PBG formation was further observed in the diffuse reflectance (DR%) spectra of the single PC films (Figure 5b,c), where a broad band following the PBG variation was clearly distinguished from the constant anatase absorption edge at 380 nm and the featureless P25 spectra at *λ* > 400 nm (Figure 5d). Deposition of the P25 top layer resulted in an increase in DR% for the P25/PC bilayers films due to the rough P25 surface morphology and a small red-shift (~20 nm) of titania’s absorption edge due to the lower (~3.0 eV) bandgap of rutile nanoparticles comprised in P25.

CoO_x_ modification resulted in a marked DR% drop, especially for CoO_x_-P25/PC406, due to the appearance of two broad absorption bands at about 450 and 750 nm, most clearly discriminated in the Kubelka–Munk absorbance spectra for CoO_x_-P25 (Figure 5d). These bands comply favorably with the strong charge transfer transitions at about 2.8 and 1.7 eV for Co_3_O_4_, corroborating the spinel formation on P25 detected by Raman. On the other hand, the CoO_x_-modified PC406 and PC499 films presented a clear DR% reduction in the range of 400–600 cm^−1^, indicating the preferential formation of amorphous CoO_x_ and/or the wider-band-gap CoO (~2.6 eV) and Co_2_O_3_ (~1.8 eV) oxides [39] in the inverse opal bottom layer, in agreement with the Raman and XPS results.

### 3.3. Photocatalytic Performance

**Figure 6:**
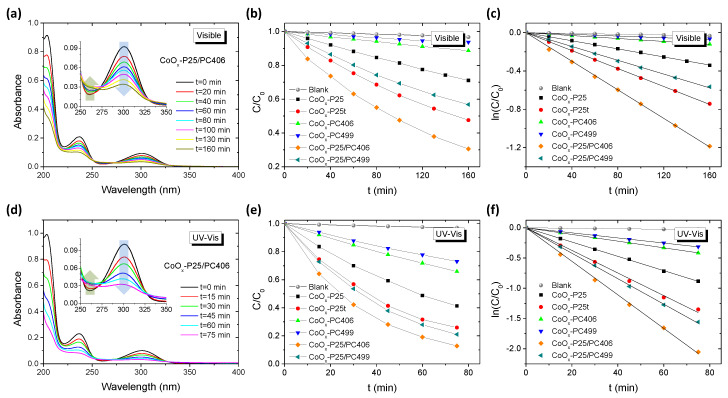
Temporal salicylic acid (SA) absorbance evolution for CoO_x_-P25/PC406 and SA photodegradation kinetics for the monolithic and bilayer CoO_x_-TiO_2_ films under (**a**–**c**) visible and (**d**–**f**) ultraviolet (UV)–visible light (Vis) irradiation. The downward and upward arrows in (**a**,**d**) depict the SA decomposition and intermediate product generation, respectively.

The photocatalytic performance of the CoO*_x_* surface-modified bilayer films and their monolithic constituents was evaluated on the photodegradation of the colorless SA water pollutant under visible and UV–Vis light, as shown in Figure 6. The SA molecules absorb in the UV range (Figure 6a,d), far below the PBGs of the PC bottom layers, excluding any contribution of slow photon enhancement by spectral overlap with the electronic absorption of the probe molecules [40]. The photodegradation experiments were conducted at acidic pH = 3 that promotes the adsorption of SA on TiO_2_ [41] and direct oxidation by titania’s valence band holes [27,28]. No significant SA degradation could be detected under direct visible and UV–Vis light, whereas illumination of the SA solution in the presence of the TiO_2_ films resulted in a continuous decrease in SA concentration (*C*) with time, monitored by the characteristic aromatic absorption band at 300 nm. In addition to the SA decomposition, a weak temporal absorbance increase was traced at the sides of the 300 nm band, at about 260 and 330 nm, reflecting the formation of intermediate degradation products in the reaction mixture (insets of Figure 6a,d), which comprise mainly dihydroxybenzoic acids and linear short-chain carboxylic acids [42,43].

**Figure 7:**
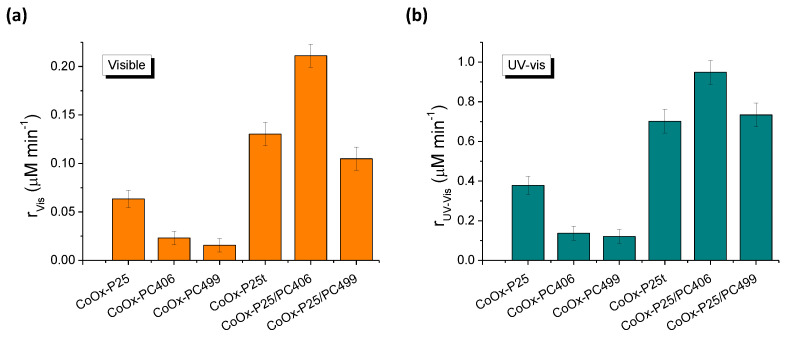
SA reaction rates of for the monolithic and bilayer CoO_x_-TiO_2_ films under (**a**) visible and (**b**) UV-Vis irradiation.

The SA photodegradation kinetics for the bilayer and reference films followed pseudo first-order kinetics under visible and UV–Vis illumination, as shown in Figure 6c,f. The apparent kinetic constants $k_{\mathrm{UV}-\mathrm{Vis}}$ and $k_{\mathrm{Vis}}$ were determined from the slopes of the linear ln(*C*/*C*_0_) vs. *t* plots, and the corresponding reaction rates r, which, for low (<mM) SA concentrations, are determined by $r=kC_{0}$, were used to quantify the films’ photocatalytic activity, independently of the initial SA concentration $C_{0}$. Figure 7 compares the obtained reaction rates $r_{\mathrm{Vis}}$ and $r_{\mathrm{UV}-\mathrm{Vis}}$ for all CoO_x_-TiO_2_ films under visible and UV–Vis illumination. All films presented significant photocatalytic activity under visible light, confirming the positive effect of CoO_x_ nanoclusters on titania’s VLA photocatalytic performance by surface states that enable visible-light activation and interfacial charge transfer that promotes charge separation and/or their cocatalyst action [14,15,16,17,18,19,20,23]. Distinct improvements of both reaction rates $r_{\mathrm{Vis}}$ and $r_{\mathrm{UV}-\mathrm{Vis}}$ were observed for the bilayer CoO_x_-P25/PC films surpassing the additive result of the constituent layers, indicating significant activity enhancement by the PC supported heterostructure.

The highest performance was observed for CoO_x_-P25/PC406, exceeding even the thick benchmark CoO_x_-P25t films that consist of much higher catalyst mass. In that case, the broad DR% of the PC406 support (Figure 5b) is expected to backscatter a substantial part of the transmitted light through the CoO_x_-P25 top layer. This would result in increased broadband visible-light harvesting by the surface modified P25 top layer, which comprises mainly Co_3_O_4_ nanoclusters that absorb light over an extended spectral range via the strong charge transfer transitions at about 450 and 750 nm (Figure 5d). At the same time, a significant part of the transmitted light through the CoO_x_-P25 layer can be utilized by the CoO_x_-PC406 substrate itself as an active photocatalytic layer. The corresponding photocatalytic contribution will be amplified, as both the low- and the high-energy edges of the PC406 bottom layer PBG, which is at about 610 nm in water, overlap with the broad CoO_x_ absorption bands, leading to slow light propagation in the inverse opal anatase skeleton and macropores of the PC substrate by “red” and “blue” slow photons, respectively [2]. On the other hand, slow photon amplification is expected to be suppressed for PC499, whose PBG is expected at about 730 nm, in agreement with its relatively lower photocatalytic activity for the monolithic CoO_x_-PCs. The longer PBG wavelength would mainly result in selective NIR Bragg backscattering of transmitted light by the PC499 support within the Co_3_O_4_ absorption range of the P25 upper layer and modest performance amplification. Combination of the photonic enhancement with the improved molecular transport within the meso-macroporous bilayer films could further promote the films’ photocatalytic efficiency for organic pollutant decomposition. Specifically, the fast diffusion of probe molecules through the inverse opal macropores that is limited by constrictions imposed by the skeleton mesopores and adsorption [44,45] can be further enhanced in the case of multilayered films, where additional transport pathways may be present at the layer interfaces according to recent results on fluid transport in multilayered inverse opals with vertically graded pore sizes [46].

**Figure 8:**
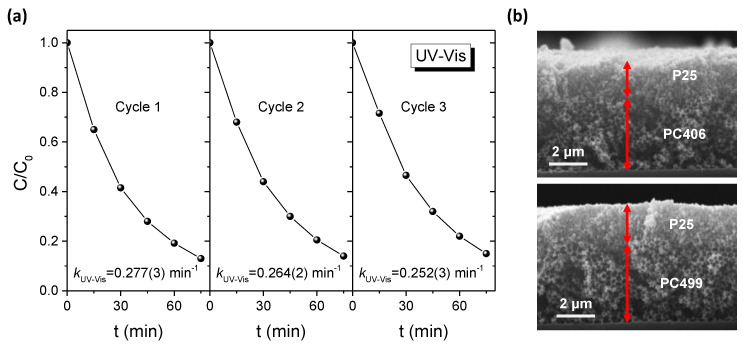
(**a**) Three consecutive SA photocatalytic runs using the one CoO_x_-P25/PC406 film under UV–Vis illumination and the apparent kinetic constants $k_{\mathrm{UV}-\mathrm{Vis}}$ derived from the corresponding ln(*C*/*C*_0_) vs. *t* plots. (**b**) SEM images of the CoO_x_-P25/PC406 and CoO_x_-P25/PC499 bilayer films after SA photodegradation tests.

The stability of the bilayer films was evaluated by three successive SA photocatalytic cycles using the same CoO_x_-P25/PC406 film under UV–Vis light, with intermediate cleaning of SA residues under additional UV–Vis illumination for 1 hr in 4 mL deionized water. A rather small decrease (3–4%) in SA degradation after 75 min of illumination was observed for the third cycle, leading to a reduction of $k_{\mathrm{UV}-\mathrm{Vis}}$ by about 9%, as shown in Figure 8a. In addition, no flaking or detachment of the films was observed after the photocatalytic experiments, while SEM measurements indicated that the films retained their bilayer structure (Figure 8b). XPS measurements on the CoO*_x_*-P25/PC406 film after SA degradation cycles indicated weak Co losses, while the Co *2p* spectra remained essentially identical to that of the initial films (Figure 4a), corroborating the chemical stability of CoO_x_ species after aqueous-phase organic pollutant photodegradation.

## 4. Conclusions

Heterostructured bilayer films with smooth interfaces were fabricated by spin-coating a highly performing mesoporous P25 titania top layer on highly ordered TiO_2_ inverse opal substrates fabricated by the coassembly of PMMA colloidal spheres with a water-soluble titania precursor. Visible-light activation of the bilayer films over a broad spectral range was performed via the surface modification of the titania layers with CoO*_x_* nanoclusters employing the Co acetylacetonate adsorption on TiO_2_ followed by post-calcination. PBG engineering of the inverse opal supporting layers to the visible-light absorption of surface CoO*_x_* oxides resulted in distinct improvement of SA photodegradation rate under visible and UV–Vis light, outperforming benchmark P25 films of higher titania mass loading. The optimal performance was accomplished by the combination of slow photon amplification for the activity of the CoO*_x_*-PC bottom layer assisted by Bragg backscattering of transmitted light that enhanced visible-light harvesting of the CoO*_x_*-P25 top layer. These results indicate that photonic crystals can be efficiently incorporated in multilayer photocatalytic films functioning as both highly effective Bragg mirrors and photocatalytically active layers.
